# An asymptomatic ectopic impacted tooth in the mandibular ramus region with congenital loss of the second lower right premolar: A rare case report

**DOI:** 10.1097/MD.0000000000041367

**Published:** 2025-02-07

**Authors:** Shencong Xu, Mingyi Ji, Chengrui Xu, Junfeng Xu

**Affiliations:** a Hospital of Stomatology, Tongde Hospital of Zhejiang Province, Hangzhou, Zhejiang, China.

**Keywords:** congenital loss of second premolar, ectopic tooth, impacted tooth, mandibular ramus

## Abstract

**Rationale:**

Ectopic teeth may be supernumerary, deciduous, or permanent and rarely occur in the general population, with an incidence of ≈0.1% to 1% according to the literature. This report presents a rare case of an ectopic impacted tooth in the mandibular ramus region, aiming to enhance clinical recognition of this condition and explore the etiology of the ectopic tooth.

**Patient concerns:**

In a patient with an asymptomatic ectopic impacted tooth in the mandibular ramus region, accompanied by congenital loss of the second lower right premolar, we found an ectopic impacted tooth in the mandibular ramus region.

**Diagnoses:**

Ectopic teeth, impacted teeth, and congenital loss of the second lower right premolar.

**Interventions:**

The impacted tooth was not extracted, and regular oral radiographs were obtained every year. For congenital loss of the second lower right premolar, we recommend implant restoration in the position of the second right mandibular premolar.

**Outcomes:**

The patient accepted our recommendation and underwent implant restoration at the position of the second right mandibular premolar.

**Lessons:**

Asymptomatic ectopic impacted teeth in the mandibular ramus region are rare. Dental development is a complex process that is influenced by a variety of factors, including genetic or acquired factors, and is regulated at many levels and in different time periods; however, it is still not clear.

## 1. Introduction

In some cases, genetic or acquired influences result in impacted teeth that are unable to erupt because of bone or soft tissue disorders. These teeth may develop in unusual positions or at a distance from their normal anatomical locations. This phenomenon is known as the ectopic tooth development. Ectopic teeth can be supernumerary, deciduous, or permanent.^[1]^ They occur rarely in the general population, with an incidence of ≈0.1% to 1% according to the literature.^[2]^ Most reported cases occur in the mandibular ramus, maxillary sinus, palate, mandibular condyle, mandibular sigmoid notch, coronoid process, orbit, nasal cavity, or through the skin.^[1–5]^ This article presents a case report of a patient who presented with congenital loss of the right lower second premolar, accompanied by an ectopic and impacted tooth in the right mandibular ramus, under the inferior edge of the sigmoid notch.

## 2. Case presentation

**Figure 1.**
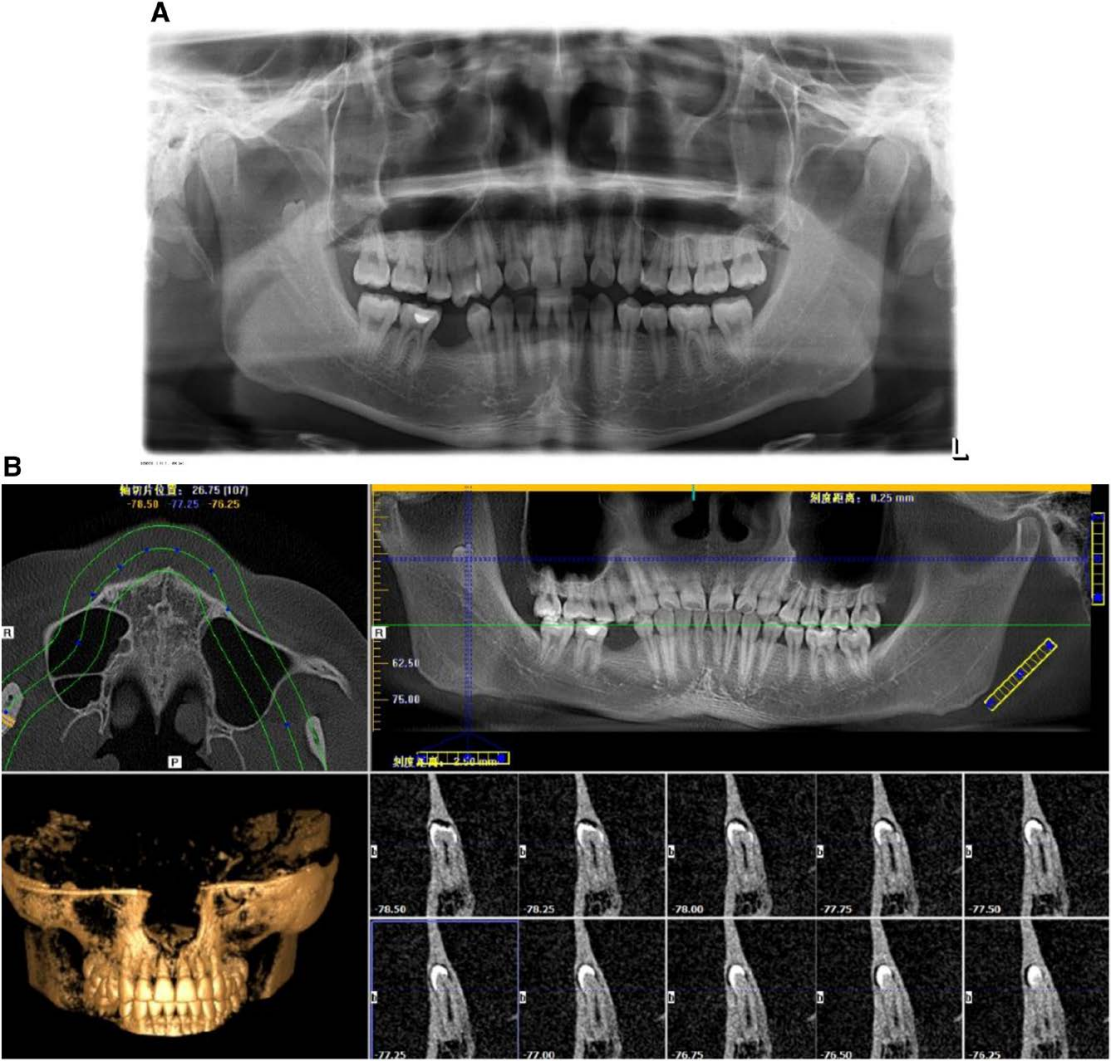
Imaging and dental clinical examination. (A and B) The panoramic radiograph and CBCT of the patient showing an impacted teeth in the Mandibular ramus. CBCT = cone beam computer tomography.

**Figure 2.**
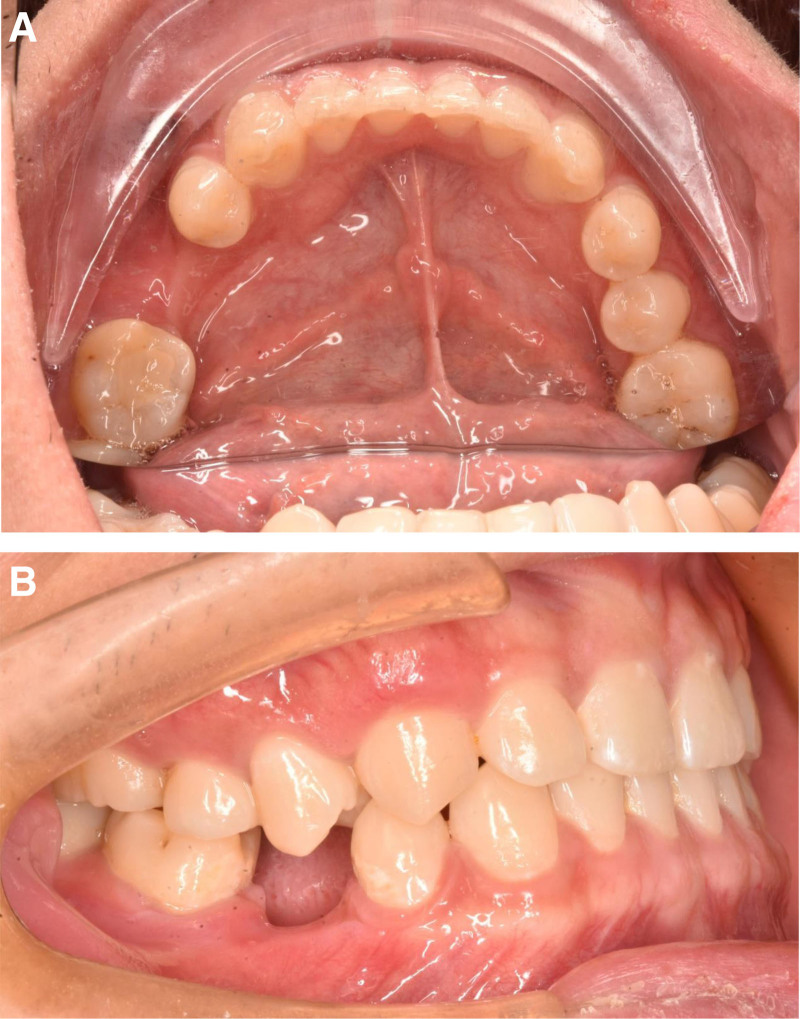
Imaging and dental clinical examination. (A and B) Oral examination showed that the second lower right premolar was missed.

**Figure 3.**
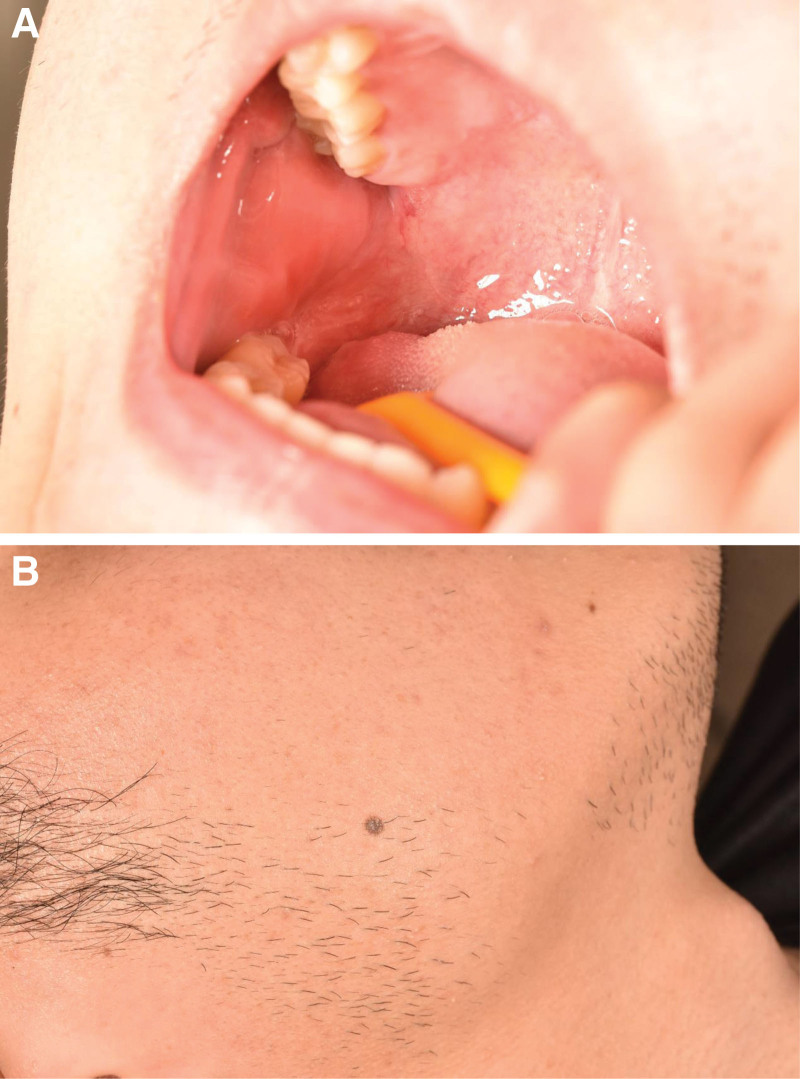
Imaging and dental clinical examination. (A and B) Oral examination showed that there were no significant swelling and pain in the examination of the right mandibular ramus.

**Figure 4.**
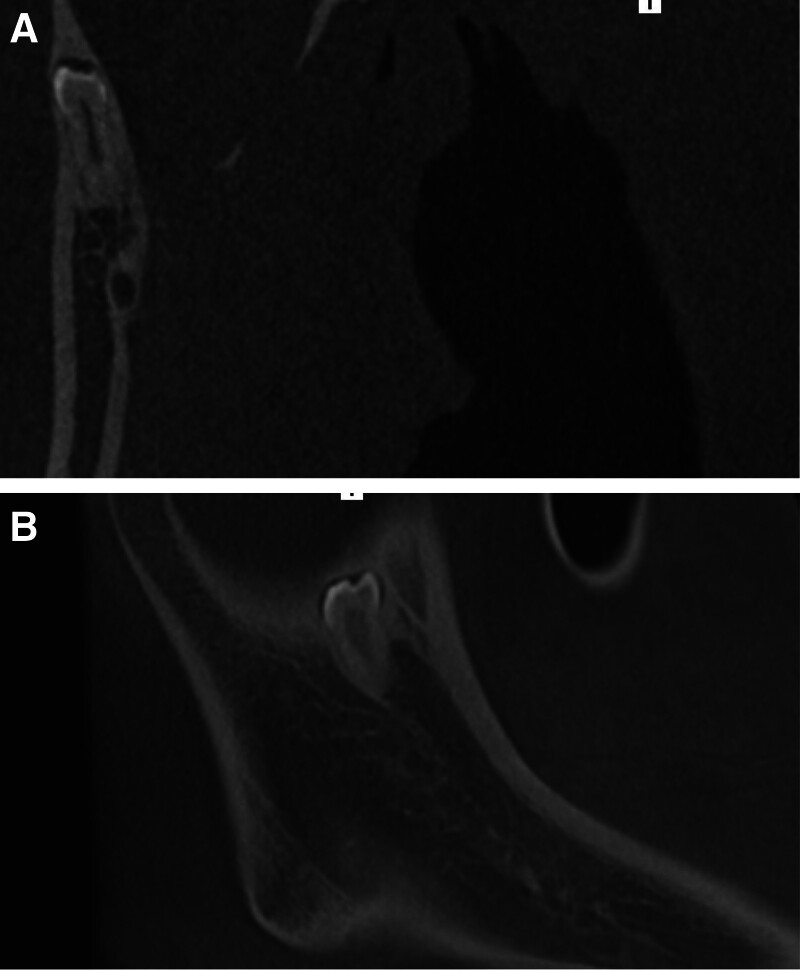
Imaging and dental clinical examination. (A and B) From the view of the coronal and sagittal position of CBCT. CBCT = cone beam computer tomography.

A 23-year-old male presented to the Department of Stomatology, Tongde Hospital, Zhejiang, China, with a history of loss of permanent teeth for 13 years. The patient reported that the permanent tooth had not erupted since the right lower deciduous molar was extracted 13 years ago. An oral panoramic radiograph (OPG) was taken, and the dentist recommended that the space of tooth 45 (#45) could be maintained and that an implant restoration could be performed after 18 years but the patient subsequently lost the oral panorama view material that he had taken 10 years ago. A further oral OPG (Fig. 1A) and cone beam computer tomography (CBCT) (Fig. 1B) were taken at Tongde Hospital, last month, and from the oral OPG and CBCT, we can see that there is an ectopic impacted tooth in the mandibular ramus region. The oral examination revealed that the second lower right premolar was absent (Fig. 2A and B). In addition, the examination of the right mandibular ramus did not reveal any obvious bulging or pain (Fig. 3A and B). From the view of the coronal and sagittal position of CBCT (Fig. 4A and B), we can see an impacted tooth in the mandibular ramus. Within the family’s medical history, no one presented with similar symptoms. The impacted tooth was not extracted, and we recommend regular oral radiographs every year. The patient accepted our recommendation and underwent implant restoration at the position of the second right mandibular premolar area.

## 3. Diagnose

Based on the patient’s history, oral examination, and radiographic findings, what are the diagnoses of the disease?

1. ectopic teeth;
2. impacted teeth;
3. congenital loss of the second lower right premolar.

## 4. Discussion

Ectopic teeth can be supernumerary, deciduous, or permanent. It is possible that such a rare case exists in the clinic, but we are not in a position to make a definitive conclusion based solely on the available medical records.

Many cases of ectopic impaction of mandibular third molars have been reported in the literature, with a prevalence of up to 23% in the latest literature,^[6]^ with slight variations in the literature and a higher prevalence in women. The prevalence of nonthird molar impacted teeth in the population is 2.63%,^[6]^ with maxillary canine teeth being the most common, accounting for ≈52%. The prevalence of impacted maxillary incisors was 45%, followed by that of maxillary canines. Ectopic mandibular third molars (EMTMs) are a relatively uncommon clinical phenomenon, characterized by their atypical location within the mandible.^[7]^ They are more frequently observed unilaterally than bilaterally.^[8]^ Wu et al^[9]^ proposed a 4-level classification system for EMTMs. Level I is observed in the upper ramus, level II in the middle ramus, level III in the mandibular angle, and level IV in the mandibular body, according to the anatomic site identified on radiographs. In the literature published by Veerabhadrappa et al,^[10]^ it was reported that among the 48 individual EMTM teeth from January 1965 to August 2020, the most common position was the subcondylar/condylar region, followed by the ramus, sigmoid notch, and angle of the mandible. The least common sites were the coronoid process and the lower border of the mandible. Some patients are diagnosed based on the presence of clinical signs and symptoms, including swelling, pain, trismus, facial asymmetry, acute infections, sinus formation, and temporomandibular joint disorder.^[9,11–13]^ Most of the patients underwent surgical treatment. Patients rarely remain asymptomatic throughout their lifetime. Furthermore, with the advent of medical radiology, panoramic radiography computed tomography, and CBCT have become invaluable tools in the diagnosis of ectopic third molars. However, a few cases are incidentally discovered during routine radiographic examinations because of other oral diseases. Due to the complexity of the disease, the etiology of EMTMs is not completely clear. One hypothesis is the developmental origin theory, which posits that the formation of ectopic teeth is attributable to the reversal of dentin, akin to that of extinct primates. In addition, the occurrence of ectopic teeth may be attributed to a deviant initial position of the third molar germs or an aberrant eruption pattern.^[14]^ A deficiency in the available space between the mandibular second molar and ramus of the mandible, or an imbalance between the base and the direction of growth of the third molar, may contribute to the abnormal positioning of the impacted third molar.^[2,15,16]^ Furthermore, Keros and Susic^[12]^ postulated that the underlying cause may be complete and primary dislocation of the third molar tooth base posteriorly from its muscle process. At present, the majority of cases documented in the literature pertain to those that exhibit clinical symptoms. However, numerous cases with no clinical symptoms have not yet been reported. Consequently, the true prevalence of EMTM may be subject to bias, particularly, in relation to those situated in the lower border, angle, and ramus of the mandible.^[17]^

In addition, the supernumerary teeth can also be impacted. Supernumerary teeth are formed from a tooth germ that exceeds the typical number for any position in the dental arch.^[18]^ These teeth may be either erupted or retained,^[19–21]^ and their prevalence varies significantly between regions, ranging from 0.2% to 5.3%.^[22]^ It has been reported that supernumerary teeth occur in both primary and permanent dentitions; however, the precise etiology remains unclear. Some authors have put forth the hypothesis that the supernumerary tooth develops from a third tooth germ that diverges from the plate of the neighboring permanent tooth bud, or may originate from the division of the permanent tooth germ itself. The morphology of the supernumerary tooth may closely resemble that of the teeth in the molar, premolar, or anterior group to which it belongs, or it may be quite different in appearance from its neighbors. Supernumerary teeth can occur in any tooth position; however, they are most frequently observed in the anterior part of the dental arch in the maxilla, representing ≈90% of the cases.^[21,23]^ Based on their location, supernumerary teeth can be classified as mesiodens (situated in the midline), paramolar (positioned buccally between the second and third molars), or distomolar (located distally to the third molar).^[24]^ The occurrence of supernumerary teeth in the mandibular ascending ramus is less common, although there are few reports in the literature on this subject. Sanghera and Jones^[25]^ documented the occurrence of fourth molars in the ramus, which were extracted via surgical intervention.

In addition, the ectopic occurrence of tooth germs may lead to the ectopic growth of teeth. In recent years, a growing body of research has indicated the possibility of a correlation between congenital absence of permanent teeth and mutations in gene loci. However, the precise mechanism by which this phenomenon occurs remains unclear. Ectopic growth and development of the permanent tooth embryo results in the failure of the permanent teeth to erupt in their normal position. This may also manifest as congenital permanent tooth loss and ectopically impacted teeth in the mouth.

Tooth development is a highly intricate process that involves reciprocal interactions between the stomodeal epithelium and the underlying cranial neural crest-derived mesenchyme.^[26,27]^ This process can be influenced by a multitude of genetic and external factors. The early stages of tooth development are dependent on the activity of major signaling pathways, which are expressed by both epithelial and mesenchymal tissues. The wingless/integrated, bone morphogenetic protein, fibroblast growth factor (Fgf), sonic hedgehog (Shh), and ectodysplasin pathways play pivotal roles in guiding tooth development, including initiation, budding, and morphogenesis.^[28]^ Initiation determines the sites in the developing jaws where teeth develop.^[29]^ Signaling by Shh and fibroblast growth factor is required from the initiation stage of tooth development onwards.^[28]^ The timing and location of Fgf-8 expression in the epithelium of the first branchial arch is concurrent with the development of dental embryos. Fgf-8 is implicated in the regulation of tooth germ initiation and tooth germ formation, and exerts a pivotal influence on the formation pattern of the first branchial arch and the position of tooth formation. Furthermore, Fgf-8 is instrumental in regulating the developmental rate and morphology of mammalian dentition.^[30]^ Thus, Fgf-8 can be regarded as the initial event in a genetic cascade that orchestrates the entire process of tooth initiation. Furthermore, additional growth factors, including bone morphogenetic proteins, Shh, and the wingless/integrate signaling pathway, play pivotal roles in a multitude of processes pertaining to embryogenesis, development, and cell differentiation. In addition to growth factors, transcription factors act as paralogous proteins that bind deoxyribonucleic acid to regulate gene expression. Various transcription factors are involved in craniomaxillofacial development. Homeotic (*HOX*) genes and neural crest cell migration play significant roles in dentition development. The *HOX* genes associated with the position of the tooth embryo are primarily distal-less homeobox, muscle segment homeobox, and paired box 9 genes. The paired box 9 gene is present in mesenchymal cells before tooth eruption, and thus serves as a marker for the position of tooth eruption. *HOX* genes also play a role in the development of tooth eruptions. Growth and transcription factors exert a significant influence on the positioning of teeth in epithelial and mesenchymal cells.^[31,32]^ Recent studies have demonstrated that apoptotic cells produce a multitude of signaling molecules that influence the behavior of surrounding cells, thereby inducing morphogenesis, migration, and alteration of cell fate.^[27]^

In recent years, several reports have shown that intraosseous tooth movement can lead to ectopic tooth movement. The migration of a tooth, defined as the movement of a partially or fully formed tooth within the bone, is rare in the literature review, and its normal eruption is prevented.^[33,34]^ When teeth are present in areas distant from the alveolar process, transmigration or migration is the term assigned to ectopia and the migrating tooth may be ectopic. According to Peck,^[35]^ tooth migration occurs only in the mandible and involves horizontal movement. Intraosseous migration of teeth is a rare phenomenon and has been rarely reported in the literature. The most common unerupted teeth with migration are the premolars, with females outnumbering males^[35–37]^ and unilateral migration outnumbering bilateral migration. Teeth tend to show mesial migration due to masticatory effort, while the exact mechanism of distal migration is unknown, although genetic and environmental factors may be relevant. Sutton^[37]^ suggested that the initial angulation of the tooth and the early loss of both the primary second molar and mandibular first permanent molar in combination with the distal migration of the mandibular premolar are contributing factors. However, Peck^[35]^ suggested that premolar migration is either incidental or idiopathic rather than genetic, unlike canine migration, based on the observation of cases of bilateral migration. In addition, Shapira and Kufinec^[25]^ found that cystic lesions and odontomas are associated with migrated teeth. In the reported literature, the ectopic mandibular second premolar may migrate to the mandibular notch,^[33]^ mandibular angle,^[38,39]^ coronoid process,^[40–42]^ ascending mandibular ramus,^[39]^ and mandibular condyle.^[41]^ The migration of a tooth may result in root resorption, tilting, sensitivity of adjacent teeth, pain, or discomfort to the patient^[43]^ but may have no effect if the migration path is far from the root of the permanent tooth.

The most recent literature on mandibular premolar migration is an 11-year follow-up case reported by Bogdanowicz et al,^[26]^ which clearly shows that the migration of the second premolar from the normal position to the mandibular ramus indirectly results in the absence of the mandibular second premolar in its normal anatomical position.

Treatment of an ectopic tooth is dependent on several factors, irrespective of its point of origin. These factors include the location of the tooth, the age and health of the patient, their financial status, and, most importantly, the presence of any discomfort symptoms. Generally, 2 treatment methods are available: conservative and surgical management. In the case of asymptomatic ectopic teeth identified on clinical radiographs, when there is no resorption of the adjacent teeth or bone, no formation of pathological structures such as cysts or odontomas, and when the risk of tooth extraction outweighs the benefits of treatment,^[26]^ a conservative approach involving regular X-ray examinations may be employed. In addition, in cases in which impacted permanent teeth exhibit migration, it may be possible to retract them to a normal position within the arch through orthodontic treatment. In contrast, for ectopic teeth with clinical symptoms, such as swelling, pain, trismus, facial asymmetry, acute infections, or other related pathological changes,^[44]^ surgical removal of the impacted tooth is the preferred course of action. This may entail an extraoral or intraoral route.^[45,46]^ The external approach can provide good exposure to the surgical site but may result in complications such as extraoral scar formation, damage to joint components, and facial nerve injury,^[46]^ and the intraoral route can avoid these problems but provides a smaller surgical site. In fact, regardless of the type of surgical approach, we should consider the location of the ectopic tooth and the surgical risks and complications, and inform the patient in detail, and obtain their consent before surgery. After surgery, careful attention should be paid to patients if a tooth is removed from the mandibular ramus region because the remaining bone is often thin and immediate fracture may occur.^[2]^

In this case, there was no discomfort or symptoms in the patient, so it was decided not to extract the impacted tooth and to have regular oral radiographs every year. For congenital loss of the second lower right premolar, we have an implant restoration in the position of the second right mandibular premolar area.

## 5. Conclusion

Asymptomatic ectopic impacted teeth in the mandibular ramus region are rare in the clinic, and most ectopically impacted teeth are found because of the clinical symptoms; therefore, the real incidence is probably higher, and it is possible that such a rare case exists in the clinic.

However, during the data collection process, we found that the patient had lost the initial oral panorama view material and had not undergone any oral OPG in the past decades, which was a great challenge for us to explore the etiology of this ectopic tooth, and we were unable to clarify whether the ectopic tooth was related to the congenital loss of mandibular second permanent premolar.

In summary, dental development is a complex process that is influenced by a variety of factors, including genetic or acquired factors, and is regulated at many levels and in different periods.^[26]^ etiologies are still unclear.

## Acknowledgments

All authors would like to acknowledge the patient and for generously permitting the use of the data in this report. The authors would also like to recognize Prof Junfeng Xu advice regarding the possible source of this ectopic tooth and gave valuable comments regarding this manuscript.

## Author contributions

**Writing—original draft:** Shencong Xu.

**Investigation:** Mingyi Ji.

**Visualization:** Chengrui Xu.

**Project administration:** Junfeng Xu.

**Writing – review & editing:** Junfeng Xu.
